# Coronary computed tomographic angiography as gatekeeper for new-onset stable angina

**DOI:** 10.1007/s12471-021-01639-7

**Published:** 2021-10-21

**Authors:** C. K. M. Boerhout, R. G. T. Feenstra, G. A. Somsen, Y. Appelman, P. Ong, M. A. M. Beijk, L. Hofstra, T. P. van de Hoef, J. J. Piek

**Affiliations:** 1 Department of Cardiology, Heart Centre, Amsterdam Cardiovascular Sciences, Amsterdam University Medical Centre, Amsterdam, The Netherlands; 2 Cardiology Centres of the Netherlands, Amsterdam, The Netherlands; 3 Department of Cardiology, Robert-Bosch-Krankenhaus, Stuttgart, Germany

**Keywords:** Chronic coronary syndromes, Coronary computed tomographic angiography, Coronary artery disease, Nonobstructive coronary artery disease, Gatekeeper

## Abstract

Patients with new-onset stable angina constitute a substantial part of the population seen by cardiologists. Currently, the diagnostic workup of these patients depends on the pre-test probability of having obstructive coronary artery disease. It consists of either functional testing for myocardial ischaemia or anatomical testing by using coronary computed tomographic angiography (CCTA) or invasive coronary angiography. In case the pre-test probability is > 5%, the current guidelines for the management of chronic coronary syndromes do not state a clear preference for one of the noninvasive techniques. However, based on the recently published cost-effectiveness analysis of the PROMISE trial and considering the diagnostic yield in patients with angina and nonobstructive coronary artery disease, we argue a more prominent role for CCTA as a gatekeeper for patients with new-onset stable angina.

## Introduction

Patients with new-onset stable angina constitute a substantial part of the population seen by cardiologists at the outpatient clinic, leading to day-to-day challenges in clinical decision-making [1]. The diagnostic workup and treatment of angina, which may be caused by coronary artery disease (CAD), aim to relief symptoms and prevent major adverse cardiac events [2]. For decades, the workup of these patients included functional testing by exercise electrocardiography (ECG), stress echocardiography, perfusion imaging by scintigraphy (single-photon emission computed tomography/positron emission tomography [SPECT/PET]) or magnetic resonance imaging (MRI) to document the location and extent of reversible wall motion abnormalities or perfusion defects [2]. Hereafter, invasive coronary angiography (ICA) was considered the gold standard to document the presence and severity of CAD. However, ICA involves costs related to hospital admission, and patients are exposed to the risk of complications related to invasive procedures.

The introduction of coronary computed tomographic angiography (CCTA) provided a save noninvasive alternative to diagnose CAD [3]. Initially, CT was used for its ability to determine the Agatston score—a reflection of the amount of calcification in the coronary vessels representing the risk of obstructive CAD in the next 10 years [4, 5]. Although this provided an adequate indication of patients who may benefit from primary prevention, with this method the severity of coronary obstruction could not be evaluated. For this purpose, CCTA was modified [6].

At first, radiation exposure in these CT protocols was relatively high and consequently, CCTA was considered less applicable in the diagnostic workup for CAD than functional tests. To date, new developments and the introduction of ECG-controlled tube current modulation, automatic dose modulation and prospective triggering allow for direct visualisation of the coronary arteries, while the radiation exposure is much lower [7]. The coronary artery lesion severity can be assessed with the Coronary Artery Disease-Reporting and Data System (CAD-RADS) classification and provides a high accuracy for the detection of both nonobstructive and obstructive CAD as compared with ICA [6, 8, 9].

CCTA’s development over the recent years has uncovered its potential as a gatekeeper diagnostic test. It is now recommended as such in patients with a low risk of obstructive CAD [2, 10]. However, we argue there is a more dominant role for CCTA in patients with new-onset stable angina who are considered for noninvasive testing by their physician at the outpatient clinic. In this paper, we provide an overview of the arguments for a simple diagnostic approach with CCTA as the gatekeeper.

## Clinical presentation of anginal symptoms

The first step in the diagnostic workup of patients with new-onset stable angina is the clinical assessment based on the patient’s symptoms, his or her previous history and the assessment of cardiovascular risk factors. Historically, treating physicians have focussed on the detection of obstructive CAD. They aim to characterise anginal symptoms and determine the risk for the presence of obstructive CAD by using the pre-test probability (based on age, symptoms and sex). The European Society of Cardiology (ESC) guidelines for chronic coronary syndromes classify symptoms due to obstructive CAD according to three hallmarks: (1) constricting discomfort across the chest, (2) exacerbation by exertion, and (3) relief by rest or use of nitroglycerin [2]. Patients have either typical angina (all three hallmarks), atypical angina (two out of three hallmarks) or nonspecific chest pain (one out of three hallmarks), and they have traditional risk factors such as older age, male sex, dyslipidaemia, history of smoking, a family history of CAD, diabetes mellitus, hypertension and increased body mass index. This classification of angina has proven its value in determining the likelihood of obstructive CAD [2]. However, it needs to be noted that most patients with CAD, especially women, present with atypical or nonspecific chest pain; 10–15% present with typical angina [11, 12].

In addition, it has become clear that obstructive epicardial disease does not explain the full spectrum of CAD causing angina [13]. In the absence of obstructive lesions, angina (as a surrogate for all symptoms due to CAD) may also be the expression of a functional disorder of the epicardial conduit artery and/or coronary microvasculature also known as angina with no obstructive coronary artery disease (ANOCA). The clinical presentation of patients with ANOCA is even more diverse [14]. Some present with similar complaints as patients with obstructive CAD; these patients often show structural changes in the microcirculation that lead to impaired vasodilatation and ischaemia during increased myocardial exertion [15].

On the other hand, patients with symptoms due to abnormal vasoconstriction—the majority of patients with ANOCA—exhibit specific features that could be distinguished from obstructive CAD (Tab. 1; [13, 14, 16]). For example, these symptoms often persist several minutes to hours and occur predominantly at rest. Patients complain of symptoms occurring at night or in the early morning hours and have a marked diurnal variation in exercise tolerance, exhibiting a reduced tolerance in the morning. Furthermore, patients with ANOCA often experience extreme fatigue and loss of energy that fluctuates over time. The risk profile for ANOCA consists of traditional risk factors and systemic inflammatory diseases. In women, it also includes female-specific factors such as premenopausal migraine or gestational hypertension and diabetes.

**Table 1:** Overview of most common clinical presentation and risk factors for obstructive and nonobstructive coronary artery disease

	Obstructive coronary artery disease	Nonobstructive coronary artery disease
*Symptoms (most common)*	Constricting discomfort around the chest	Constricting discomfort around chest and/or shortness of breath
*Clinical features*	Exacerbated by exertion	Occurring at rest (predominantly at night or in early morning)
	Radiation to shoulder, neck, or jaw	Marked diurnal variation in exercise tolerance (reduced in morning)
		Hyperventilation can precipitate episode
*Relieving factors*	Rest and/or sublingual nitroglycerin	No or suboptimal relief by sublingual nitroglycerin
*Duration*	Approximately 1–15 min	Minutes to hours
*Risk factors*	Traditional risk factors** (**hypertension, DM, smoking, dyslipidaemia, obesity**)**	Traditional risk factors
	Familial predisposition	Familial predisposition
	Male gender	Female gender
	Older age	Chronic inflammatory disease (i.e. rheumatoid arthritis)
		Female-specific (i.e. premenopausal migraine, pregnancy-related hypertension and DM)
		Psychological stress

*DM* diabetes mellitus

In conclusion, there is a wide spectrum of symptoms related to both obstructive and nonobstructive CAD beyond the traditional presentation of typical angina. This overlap of symptoms among patients with obstructive and nonobstructive CAD indicates that symptoms are a poor indicator of the underlying coronary substrate. Therefore, CCTA may serve as an additional tool to guide medical therapy for patients with new-onset angina [13, 17, 18].

## Diagnostic approach in patients with suspected obstructive coronary artery disease

After initial assessment of the patient’s clinical presentation, treating physicians may suspect obstructive CAD based on the pre-test probability (age, sex and symptoms) and risk factors. According to the guidelines for the management of chronic coronary syndromes, patients with a pre-test probability ≤ 5% can be treated conservatively without additional diagnostic testing because of the very low risk for the presence of obstructive CAD. However, for the majority of patients—those with a pre-test probability > 5%—additional testing may be considered and either anatomical testing (to document extent and severity of CAD) or functional testing (to document myocardial ischaemia) can be used to determine further management. It is advocated to use CCTA for low-risk patients and functional testing for high-risk patients. However, the randomised PROMISE and SCOT-HEART trials concluded that both strategies are equivocal for patient management [17].

These studies were the first trials in which both strategies were compared in patients with new-onset angina that was suspected to be caused by obstructive CAD [11, 12]. At the 2‑year follow-up point, the results of the randomised PROMISE trial, which involved over 10,000 patients with stable angina and a low to intermediate risk of CAD, indicated that an anatomical approach using CCTA was non-inferior to a functional testing approach (MRI, PET/SPECT or stress echocardiography) with respect to the composite endpoint of death, myocardial infarction, hospitalisation for unstable angina or major procedural complications [11]. The SCOT-HEART trial even reported a 38% reduction in the incidence of nonfatal myocardial infarction at 5 years follow-up by a CCTA-based approach in comparison with a functional approach [12]. A possible explanation for this observation is related to the initiation of preventive medical treatment as a result of early visualisation of subclinical atherosclerosis by CCTA. On the other hand, the functional arm in the SCOT-HEART trial consisted largely of exercise ECG as the functional test, and the results are therefore less applicable to daily practice.

More recently, the cost-effectiveness analysis of the PROMISE trial, which was reported after the publication of the 2019 ESC Guidelines, showed that an anatomical approach results in lower costs than functional testing. The cost-effectiveness was the result of improved discrimination between nonobstructive and obstructive CAD and the ability to tailor medical therapy [19]. In addition, a subanalysis of the PROMISE trial showed a significant improvement of patient compliance to statin therapy in the CCTA arm regardless of the CCTA outcome; the lower adherence to statin treatment in the group guided by a functional test (67% in the functional group vs 86% in the CCTA group) was associated with a higher rate of major adverse cardiovascular events during 2 to 5 years of follow-up in the functional group [19].

These results indicate an important advantage of CCTA over functional testing in patients with new-onset anginal symptoms. Moreover, it is important to note that recent studies, such as the ISCHEMIA trial, indicate that only patients with severe symptoms benefit from revascularisation, while patients with mild symptoms can be safely treated medically [20, 21]. Therefore, it is essential that cardiologists refrain from their so-called ‘oculostenotic reflex’—the reflex to revascularise any visible stenosis—that is potentially triggered by using CCTA as a gatekeeper and that ICA should be reserved for patients who do not respond to the initiated medical treatment.

Based on these considerations, a strategy with early CCTA as a gatekeeper could be more practical and a simpler alternative to the aforementioned recommendations of the ESC. Referral for ICA may be restricted to patients who do not respond to medical therapy, who are considered at high risk based upon anatomical features such as left main CAD and/or proximal lesions of the left anterior descending coronary artery or who have an impaired left ventricular function.

### CCTA in patients with suspected nonobstructive coronary artery disease

In addition to the benefits of early detection of obstructive CAD, early detection of nonobstructive CAD in patients with new-onset stable angina is also beneficial. Evidence is emerging regarding the relevance of stratified medical therapy in ANOCA patients. In a recent study, ANOCA patients were found to have more severe anginal complaints and worse quality of life than matched patients with angina related to obstructive CAD at 6 months of follow-up [22]. Moreover, the recently published CorMicA study showed that early targeted treatment in patients with ANOCA improves symptoms and quality of life significantly at 6 months and 1 year of follow-up [18].

Although the benefits of early targeted treatment are evident, in most cases sufficient targeted therapy is only established after ICA including intracoronary functional testing. Although intracoronary functional testing is in general safe and its value is increasingly acknowledged, expertise and experience are prerequisites for the safe and correct interpretation of the procedures. Consequently, the test is performed in specialised centres only and widespread application is restricted. However, adequate assessment of the patient’s symptoms and the information provided by CCTA to exclude obstructive CAD may limit the number of patients who require ICA with intracoronary functional testing. After the first clinical assessment, the treating physician evaluates the patient’s symptoms as angina or angina equivalent (Tab. 1) and steers towards targeted therapy for different ANOCA domains: abnormal vasoconstriction, abnormal vasodilatation or a combination of both domains.

As described earlier, patients with abnormal vasoconstriction frequently exhibit atypical symptoms (Tab. 1). They benefit from targeted treatment that inhibits the pathological process of abnormal vasoconstriction and that may include long-acting nitrates or calcium channel blockers [23]. In contrast, beta blockers—frequently used to reduce angina symptoms in the first-line management of obstructive CAD—may provoke vasoconstriction and aggravate anginal burden [13]. Patients with abnormal vasodilatation, which is not frequently encountered in nonobstructive CAD, due to structural changes in the microcirculation often have similar anginal complaints as those with obstructive CAD. The aim of the therapy for these patients is similar to that of obstructive CAD, i.e. reducing the oxygen demand of the myocardium. Only those patients who do not respond adequately to this regimen of antianginal therapy that is based upon clinical judgement and the result of CCTA, should undergo intracoronary functional testing.

In conclusion, with the correct interpretation of ANOCA symptoms and by using early CCTA, patients can be offered tailored treatment at an early stage, which may reduce future medical expenses (hospital admissions and costs related to invasive and noninvasive diagnostics). ICA with intracoronary functional testing should be limited to patients who do not respond to medical therapy to assess abnormalities in the vascular domains, i.e. epicardial and/or microvascular vasoconstriction and impaired microvascular vasodilation, and to tailor medical treatment. Finally, ICA with intracoronary functional testing may also be useful for patients who require a definite diagnosis after sometimes many years of uncertainty regarding their complaints and numerous hospital admissions. Altogether, this diagnostic workup allows for appropriate selection of patients who may benefit from ICA with intracoronary functional testing, which can be performed in a limited number of specialised, high-volume centres.

## Diagnostic pathway with CCTA as a gatekeeper

**Fig. 1:**
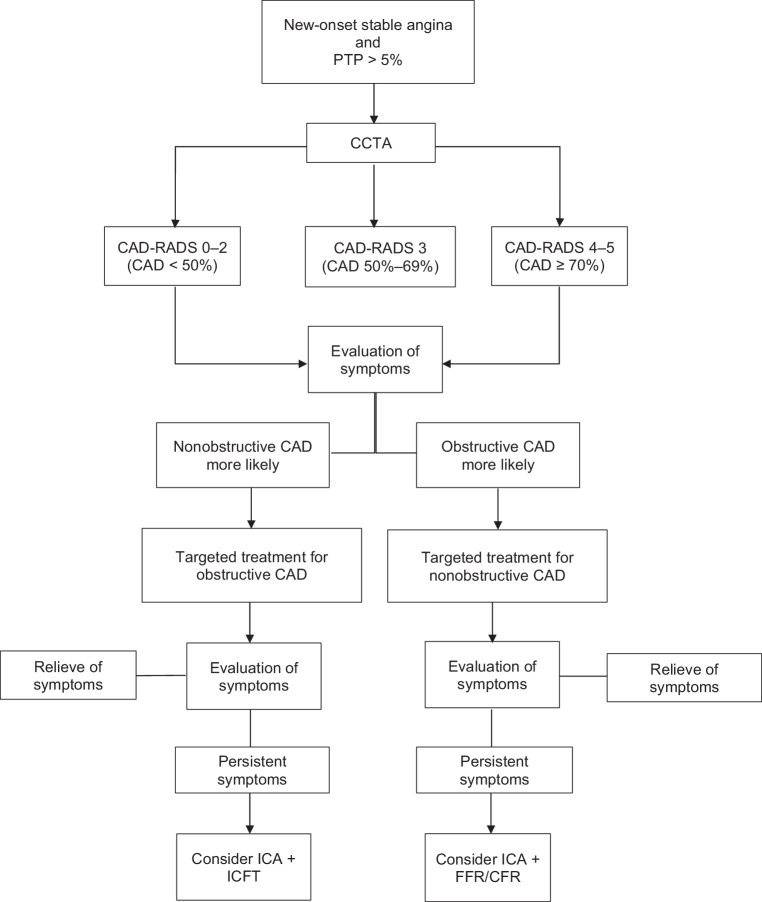
Flowchart for diagnostic approach of patients with new-onset angina with CCTA as gatekeeper. *PTP* pre-test probability, *CCTA* coronary computed tomographic angiography, *CAD-RADS* Coronary Artery Disease-Reporting and Data System, *CAD* coronary artery disease, *ICA* invasive coronary angiography, *ICFT* intracoronary functional testing, *FFR* fractional flow reserve, *CFR* coronary flow reserve

Based on the aforementioned arguments, we argue that CCTA should serve as a gatekeeper in patients with new-onset stable angina who are considered for noninvasive testing based upon a pre-test probability > 5%. A stepwise approach using CCTA as a gatekeeper is depicted in Fig. 1. The interpretation of the patient’s symptoms and direct visualisation of the coronary arteries by CCTA allow for discrimination of anginal symptoms due to nonobstructive CAD (CAD-RADS score 0–2) or obstructive CAD (CAD-RADS score 4–5). As stated earlier, this will result in a stratified therapy for both entities. In patients with mild obstructive lesions (CAD-RADS score 3) both strategies may be indicated depending upon the interpretation of the patient’s complaints. After re-evaluation of symptoms, therapy may be switched.

As a result of early stratification and targeted medical therapy, ICA is reserved for patients who remain symptomatic in both arms, thus reducing the false-positive results of CCTA [21]. In these patients with obstructive CAD, one could argue to use functional testing to assess the extent and/or location of myocardial ischaemia prior to ICA. A more practical alternative, however, is direct referral for ICA and the use of intracoronary physiological parameters (e.g. fractional flow reserve and coronary flow reserve), which allows for selecting the lesions that are responsible for the patient’s complaints. This is in particular relevant for lesions in neighbouring vascular territories, as culprit lesions are difficult to distinguish by noninvasive functional tests [24].

In this respect, it is important to note that the proposed diagnostic strategy can only be implemented if there is sufficient availability of CT scans. However, a randomised comparison of a diagnostic workup using CT scans and a targeted conservative approach, as proposed in this opinion paper, versus standard clinical care is currently lacking. Such a trial could provide important information regarding clinical endpoints and the cost-effectiveness of both diagnostic pathways.

In conclusion, the proposed diagnostic workup provides a simple and practical diagnostic strategy in which CCTA plays a more prominent role in order to improve patient care, reduce the number of unnecessary ICAs and interventions, and lower medical expenses.

### Potenzial limitations

CCTA has developed into a more rapid diagnostic technique than other noninvasive functional tests such as PET/SPECT scintigraphy and MRI. Despite technical refinements of CCTA, one still has to consider the radiation exposure (< 2 mSv) for patients, especially when considering a more liberal use of this technique [25]. Moreover, CCTA requires the use of contrast and evaluation of renal function prior to the procedure. On the other hand, it is important to note that the proposed diagnostic workup may prevent unnecessary ICA in both arms and consequently reduce avoidable exposure to renal toxic contrast in this patient population. It is obvious that a more liberal use of CCTA generates additional costs related to required personnel and equipment, but this diagnostic approach may also lead to a reduction of unnecessary ICA and its inherent costs.

A more widespread use of CCTA will be hampered by the availability of CT scanners and required medical staff to perform and analyse these procedures. In this respect, recent developments in the field of artificial intelligence may facilitate the proposed diagnostic workup on a broader scale, which may reduce current medical costs [26].

Lastly, it is important to note that the current literature does not provide evidence for the use of CCTA in patients with a history of cardiovascular diseases, such as prior established CAD, prior cardiac interventions (coronary artery bypass grafting or percutaneous coronary intervention) and/or arrhythmias, as they were excluded from the major trials.

## Conclusion

This opinion paper advocates a more prominent role of CCTA in the diagnostic workup of patients with new-onset stable angina. This straightforward approach supports tailored medical therapy in patients with either obstructive or nonobstructive CAD. Moreover, it may avoid unnecessary ICA by restricting its use to those patients who do not respond to initial medical therapy. In these patients, CCTA provides a better selection of patients who may require revascularisation or who should be considered for invasive coronary functional testing.
